# How to Achieve Sufficient Endogenous Insulin Suppression in Euglycemic Clamps Assessing the Pharmacokinetics and Pharmacodynamics of Long-Acting Insulin Preparations Employing Healthy Volunteers

**DOI:** 10.3389/fphar.2022.899798

**Published:** 2022-07-22

**Authors:** Hui Liu, Ting Li, Hongling Yu, Jiaqi Li, Huiwen Tan, Yerong Yu

**Affiliations:** ^1^ General Practice Ward, General Practice Medical Center, Sichuan University West China Hospital, Chengdu, China; ^2^ Department of Endocrinology and Metabolism, Sichuan University West China Hospital, Chengdu, China

**Keywords:** endogenous insulin suppression, euglycemic clamp, healthy volunteers, pharmacokinetics, pharmacodynamics

## Abstract

The therapeutic effect of basal insulin analogs will be sustained at a rather low insulin level. When employing healthy volunteers to assess the pharmacokinetics (PK) and pharmacodynamics (PD) of long-acting insulin preparations by euglycemic clamp techniques, endogenous insulin cannot be ignored and sufficient endogenous insulin inhibition is crucial for the PD and/or PK assessment. This study aimed to explore a way to sufficiently inhibit endogenous insulin secretion. Healthy Chinese male and female volunteers were enrolled. After a subcutaneous injection of insulin glargine (IGlar) (LY2963016 or Lantus) (0.5 IU/kg), they underwent a manual euglycemic clamp for up to 24 h where the target blood glucose (BG) was set as 0.28 mmol/L below the individual’s baseline. Blood samples were collected for analysis of PK/PD and C-peptide. The subjects fell into two groups according to the reduction extent of postdose C-peptide from baseline. After matching for the dosage proportion of Lantus, there were 52 subjects in group A (C-peptide reduction<50%) and 26 in group B (C-peptide reduction≥50%), respectively. No significant difference was detected in age, body mass index, the proportion of Latus treatment and female participants. A lower basal BG was observed in group B compared to group A (4.35 ± 0.26 vs*.* 4.59 ± 0.22 mmol/L, *p* < 0.05). The clamp studies were all conducted with high quality (where BG was consistently maintained around the target and exhibited a low variety). The binary logistic regression analysis indicated low basal BG as an independent factor for the success of sufficient endogenous insulin suppression. In conclusion, setting a lower sub-baseline target BG (e.g., 10% instead of 5% below baseline) might be an approach to help achieve sufficient endogenous insulin suppression in euglycemic clamps with higher basal BG levels (e.g., beyond 4.60 mmol/L).

## Introduction

Recently a continued global increase in diabetes prevalence and a significant global challenge to the health and well-being of individuals, families, and societies were confirmed by the IDF Diabetes Atlas 10th edition (International Diabetes Federation, 2021). Insulin is one of the most powerful drugs to normalize blood glucose (BG) in the treatment of diabetes mellitus. Benefitting from the development of pharmaceutical technology, the new generation of basal insulin formulations present growingly favorable pharmacokinetic and pharmacodynamic properties, including flatter, peakless action profiles, less inter- and intra-patient variability, and longer duration of activity (Frias and Frias, 2017) which is proven to significantly mitigate the incidence of hypoglycemia (especially during the night) compared with the previous generation. For the approval of a novel or biosimilar basal insulin preparation, the Food Drug Administration (FDA) (Food And Drug Administration, Center For Drug Evaluation And Research, 2008; Food And Drug Administration, Center For Drug Evaluation And Research, 2019) and the European Medicines Agency (EMA) (European Medicines Agency, 2015) recommend applying euglycemic glucose clamp to evaluate the pharmacokinetic exposure and pharmacodynamic activity of insulin products as well as assessing the safety and tolerability. Either the healthy volunteers or patients with type 1 diabetes mellitus (T1DM) are eligible for clamp studies (European Medicines Agency, 2015). Conflicting clamp results of long-acting insulin were reported. For example, enrollment of the healthy was questioned by some investigators (Porcellati et al., 2015) for that some PK estimates in healthy volunteers (Linnebjerg et al., 2015) differed from those in type 1 diabetic patients (Lepore et al., 2000; Porcellati et al., 2007); the time-action profile of insulin glargine (IGlar) was reported with the characteristics of being flat in healthy volunteers (Lepore et al., 2000; Scholtz et al., 2005), and was described to be up-and-down in diabetic patients (Luzio et al., 2006; Klein et al., 2007; Lucidi et al., 2011). In addition, endogenous insulin secretion in patients with T1DM is always negligible. Therefore, subjects with T1DM are generally thought to be more appropriate for the determination of the pharmacodynamic activity of long-acting insulin. However, many issues should be taken into consideration when enrolling patients with T1DM: 1) subjects with T1DM usually exhibit higher inter-individual variability (Kapitza et al., 2020) than healthy subjects (Li et al., 2021), which might require a larger sample size; 2) insulin resistance is not only a characteristic feature of T2DM but also consistently found in T1DM (Yki-jarvinen & Koivisto, 1986; Cleland et al., 2013), and variable extents of insulin sensitivity require strict glucose infusion adjustment; 3) progression of devastating microvascular complications, including nephropathy, retinopathy, and peripheral sensory and autonomic neuropathy can be caused by diabetes (American Diabetes Association Professional Practice Committee, 2022). High prevalence rates of microvascular complications were detected even in young adults with T1DM (James et al., 2014), which would increase the difficulty of subject recruitment and management; 4) moreover, prescribed medicine for comorbidity sometimes may affect the assessment of study insulin; 5) an additional run-in period of up to 6 h is usually required before clamping for the normalization of BG (Porcellati et al., 2019; Linnebjerg et al., 2020a; Heise et al., 2020). From the above considerations, healthy volunteers are considered to be another eligible option.

Since the therapeutic effect of basal insulin could be sustained at a rather low level for a rather long period, endogenous insulin secreted by healthy individuals cannot be ignored. A consistent and adequate endogenous insulin suppression is crucial for the accurate time-action and/or time-concentration profiles of long-acting insulin (Heise et al., 2016). C-peptide is always regarded as a marker of endogenous insulin secretion. A C-peptide reduction of 60% was observed when the insulin level approximately reached 100 mU/L by continuous infusion (Defronzo et al., 1979). Without infusing a large amount of exogenous insulin to establish hyperinsulinemia, different extents of C-peptide reduction, such as >30% (Heinemann et al., 2000), 35% (Starke et al., 1989), approximately 40% (Sorensen et al., 2010), >50% (Heinemann et al., 1999; Scholtz et al., 2005) were reported. When the C-peptide level was inhibited to at least 50% of the baseline, the rise of glucose infusion rate (GIR) was considered to be independent of contribution from endogenous insulin (Doberne et al., 1981). Hence, a C-peptide reduction of more than 50% is widely recognized as sufficient endogenous insulin suppression. Many clamp studies have reported continuous endogenous insulin suppression (Heinemann et al., 2000; Zhang et al., 2017; Drai et al., 2022), while enhancement of adequate endogenous insulin inhibition is still needed.

In this study, our primary aim is to seek a way to assure adequate endogenous insulin inhibition in euglycemic clamps evaluating the PK/PD of long-acting insulin in healthy volunteers. Second, since C-peptide is always used to correct endogenous insulin in the pharmacokinetic assessment of exogenous insulin in the absence of specific assays (Brunner et al., 2000; Scholtz et al., 2005; Plum-Morschel et al., 2022), we try to seek the effect of different extents of C-peptide reduction on the pharmacokinetic and pharmacodynamic assessment.

## Materials and Methods

### Subjects

This study enrolled healthy Chinese men and women aged 18–40 years old (inclusive at screen visit), with body mass index (BMI) 18.0–28.0 kg/m^2^, fasting glucose <100 mg/dl, and a normal glucose response to a 75 g OGTT (2 h PG < 140 mg/dl). No abnormalities were detected in clinical and laboratory assessments, including past medical history, vital signs (blood pressure, body temperature, heart, and respiratory rate), liver and renal function, a 12-lead electrocardiogram, a complete blood count, and a urinalysis to certify the overt health of the subjects.

The study was approved by the ethics committee of West China Hospital of Sichuan University and conducted in accordance with the Declaration of Helsinki and Good Clinical Practice guidelines. All subjects provided written informed consent prior to participation in the study after receiving detailed information about this study.

### Study Design

All enrolled subjects underwent a euglycemic glucose clamp lasting for up to 24 h after a 0.5-IU/kg IGlar injection. All the participants were restrained from strenuous exercise, smoking, alcohol, or caffeinated drinks. Since a C-peptide suppression of more than 50% is widely recognized as sufficient endogenous insulin suppression, the subjects fell into two groups based on the ratio of C-peptide reduction from baseline (group A: C-peptide reduction <50%; group B: C-peptide reduction ≥50%). The data were collected from a clinical trial (NCT03555305) (Liu et al., 2021), which was conducted to evaluate the pharmacokinetic and pharmacodynamic equivalence of an IGlar biosimilar (LY2963016) manufactured by Eli Lilly to Lantus manufactured by Sanofi.

### Euglycemic Clamp Procedure

At the dosing visit, an overnight fast of at least 8 h must be guaranteed prior to IGlar administration. Two catheters were placed into the antecubital vein and the hand vein, respectively, for 20% dextrose infusing and blood sampling correspondingly. The baseline of BG was defined as the mean of three BG measurements at 30, 20, and 10 min prior to the IGlar administration. A single dose (0.5 IU/kg) of Lantus or LY2963016 was subcutaneously injected into a lifted abdominal skinfold. A 0.1-ml blood sample was collected from the hand vein which was continuously heated in a warm blanket (55 ± 5 °C) at an interval of 10–20 min for the analysis of BG levels. A variable GIR was frequently adjusted and timely recorded to maintain the BG around 0.28 mmol/L below the baseline and represent the pharmacodynamic activity of IGlar, respectively.

The BG levels were immediately determined in whole blood at the bedside by a glucose analyzer (Biosen C_line, EKF Diagnostics, Barleben, Germany) with the glucose oxidase method. The measuring range of this method was 0.5–50 mmol/L with a high precision of 1.5%. The clamp would end if no exogenous glucose infusion was required for at least 0.5 h.

### Pharmacokinetic Sampling and Bioanalytical Analysis

A 6-ml blood sample for analysis of C-peptide and total insulin levels were collected before drug administration (0.5 and 0 h before dosing) and every 0.5–3 h throughout the clamp as described before (Liu et al., 2021). The whole blood sample was drawn into a serum tube and gently mixed by inversion (>5 times). Then the sample was required to clot for 30–60 min at room temperature. The tube was centrifuged at 1,500–2000g for 15–20 min to separate serum. The serum was transferred without red blood cells contained, and frozen at -70 °C until shipment for analysis. The concentration of total insulin was determined by a validated radioimmunoassay method (Kuerzel et al., 2003; Shen et al., 2019) with a quantification range of 50–2000 pmol/L at WuXi AppTec Co., Ltd. in Shanghai, China. The exogenous IGlar level was calculated by subtracting the endogenous insulin based on C-peptide using Owens’s method (Owens, 1986). The C-peptide concentration was analyzed by a direct chemiluminescent technology in Covance Laboratory (Shanghai, China) with a quantification range of 0.07–352 ng/ml.

If a measured value of total insulin or C-peptide was below the lower limit of quantification, it would be included in statistics as half of the limit. A calculated value of IGlar below zero after C-peptide correction would be excluded from the PK statistics. The primary PK parameters included the area under the curve (AUC) of IGlar from time 0–24 h (AUC_IGlar, 0–24 h_) and the maximum IGlar (IGlar_max_) corrected by C-peptide. Other PK parameters included AUC of IGlar from 0 to 12 h (AUC_IGlar, 0–12 h_) and time to IGlar_max_ (tIGlar_max_).

Individual GIR values were smoothed by a locally weighted scatterplot smoothing technique. The maximum GIR (GIR_max_) and the total amount of glucose infused throughout the clamp (AUC_GIR, 0–24 h_) were defined as primary PD parameters. Secondary PD parameters included time to GIR_max_ (tGIR_max_) and AUC of GIR from 0 to 12 h (AUC_GIR, 0–12 h_).

### Clamp Quality Assessment

The quality of clamp studies was assessed based on the all BG measurements throughout the clamp and the target BG in the individual clamp as previously described (Benesch et al., 2015): 1) coefficient of variation of BG (CVBG), 2) mean absolute difference of every measured BG from target level in the individual clamp.

### Statistical Analyses

A previous study showed (Liu et al., 2021) that for IGlar_max,_ and AUC_IGlar, 0–24 h_, geometric least-squares mean ratios (90% confidence interval) of LY2963016 to Lantus were 0.961 (0.887-1.04) and 0.941 (0.872-1.01), respectively, and dosage proportion of Lantus was significantly different between two groups (Table 1), therefore, matching for dosage proportion of Lantus was performed. Normally distributed data were expressed as arithmetic mean with standard deviation and analyzed by unpaired Student’s t-test. Non-Normal distributed parameters were expressed as median with interquartile range and analyzed by Mann Whitney test. Categorical variables were compared using the Chi-square test. The binary logistic regression analysis was performed with SPSS 22.0 software. For all tests, a significance level of 5% (two-sided) was used.

**TABLE 1:** Demographics of group A and group B before and after LY2963016 and Lantus dose proportion matching.

	Group A	Group B	P
Before matching for dose proportion of LY2963016 and Lantus			
N	86	28	-
Age (year)^a^	25.3 ± 2.7	24.5 ± 1.6	0.145
Height (cm)^a^	164.7 ± 8.9	164.8 ± 7.3	1.000
Weight (kg)^a^	60.0 ± 11.5	57.1 ± 7.3	0.215
BMI (kg/m^2^)^a^	21.9 ± 2.4	21.0 ± 1.8	0.070
Dose of exogenous insulin (IU)^a^	30.0 ± 5.7	28.8 ± 3.8	0.291
N of Lantus treatment (N,%)	40, 46.5%	18, 64.3%	0.102^b^
Dosage proportion of Lantus (%)	48.6%	63.9%	<0.001^b^
Female proportion (%)	58.1%	53.6%	0.671^b^
After matching for dose proportion of LY2963016 and Lantus			
N	52	26	-
Age (year)^a^	25.4 ± 2.6	24.3 ± 1.5	0.051
Height (cm)^a^	161.9 ± 7.0	164.4 ± 7.5	0.140
Weight (kg)^a^	56.7 ± 8.9	56.3 ± 7.0	0.837
BMI (kg/m^2^)^a^	21.6 ± 2.3	20.8 ± 1.8	0.136
Dose of exogenous insulin (IU)	28.3 ± 4.5	28.3 ± 3.6	1.000
N of Lantus treatment (N,%)	32, 61.5%	16, 61.5%	1.000^b^
Dosage proportion of Lantus (%)	61.2%	60.5%	0.758^b^
Female proportion (%)	71.2%	57.7%	0.234^b^

BMI, body mass index.

a Arithmetic mean ± standard deviation, the difference was detected by unpaired Student’s t-test.

b Difference was detected by the chi-square test.

## Results

### Demographics and Disposition

A total of 114 subjects were eligible for this study. According to the extent of postdose C-peptide reduction, there were 86 subjects in group A (C-peptide reduction<50%) and 28 in group B (C-peptide reduction≥50%). The percentages of Lantus dosage significantly differed between the two groups (*p* < 0.001, Table 1). To minimize the effect of drug difference (either LY2963016 or Lantus) on the PK/PD evaluation, matching for the dose proportion of Lantus was performed. Thereafter, 52 and 26 subjects were enrolled in groups A and B, respectively. No statistical difference was detected in age, height, weight, BMI, total exogenous IGlar, or dosage proportion of Lantus between the two groups (Table 1).

### Euglycemic Clamp Statistics and C-Peptide Levels

**FIGURE 1:**
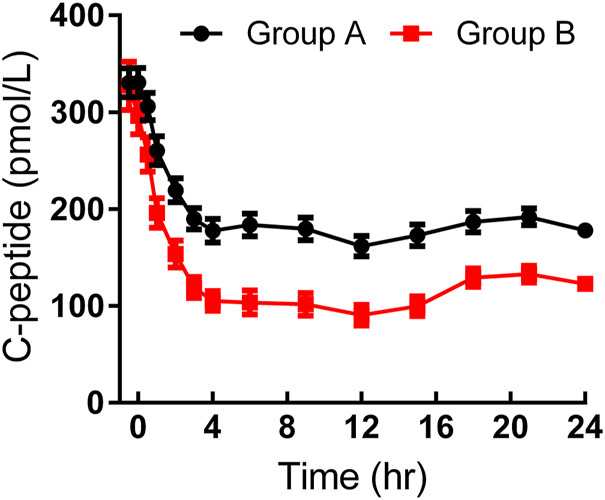
Time-profiles of serum C-peptide throughout the euglycemic clamp in groups A and B, respectively (Mean ± SEM).

As shown in Table 2, basal and target BG levels were higher in group A than those in group B. BG was continuously maintained around the target level in both groups. The CVBG and mean absolute difference of BG from the target were relatively lower than those previously reported (Scholtz et al., 2003; Hordern et al., 2005). The basal C-peptide and insulin levels were both comparable, while a stronger C-peptide reduction was observed in group B (Figure 1). No significant difference was detected in HOMA-IR.

**TABLE 2:** Euglycemic Clamp Statistics and C-peptide levels.

	Group A (N = 52)	Group B (N = 26)	P
Basal BG (mmol/L) ^a^	4.59 ± 0.22	4.35 ± 0.26	<0.001
Target BG (mmol/L) ^a^	4.31 ± 0.22	4.07 ± 0.26	<0.001
‘Clamped’ BG (mmol/L) ^a^	4.31 ± 0.21	4.09 ± 0.23	<0.001
CVBG (%)^a^	2.98 ± 0.79	3.36 ± 0.70	0.041
Mean absolute difference from target (mg/dl) ^a^	1.85 ± 0.42	2.05 ± 0.58	0.084
Basal C-peptide (pmol/L) ^a^	331 ± 106	312 ± 101	0.728
Mean postdose C-peptide (pmol/L) ^a^	201 ± 74	134 ± 50	<0.001
C-peptide reduction (%)^a^	39.5 ± 6.7	57.4 ± 5.5	<0.001
Basal insulin (pmol/L) ^a^	50.0 ± 27.0	49.0 ± 25.3	0.880
*F* (%)^a^	14.6 ± 5.1	15.5 ± 5.7	0.513
HOMA-IR (pmol/L×mmol/L) ^a^	10.3 ± 5.8	9.5 ± 5.1	0.563

BG, blood glucose; CV, coefficient of variation; Basal C-peptide, the mean of C-peptide levels at -0.5 and 0 h predose; Basal insulin, the mean of insulin levels at -0.5 and 0 h predose; *F*, ratio of basal insulin to basal C-peptide; HOMA-IR, homeostasis model assessment of insulin resistance.

a Arithmetic mean ± standard deviation, the difference was detected by unpaired Student’s t-test.

### Pharmacokinetics and Pharmacodynamics

**FIGURE 2:**
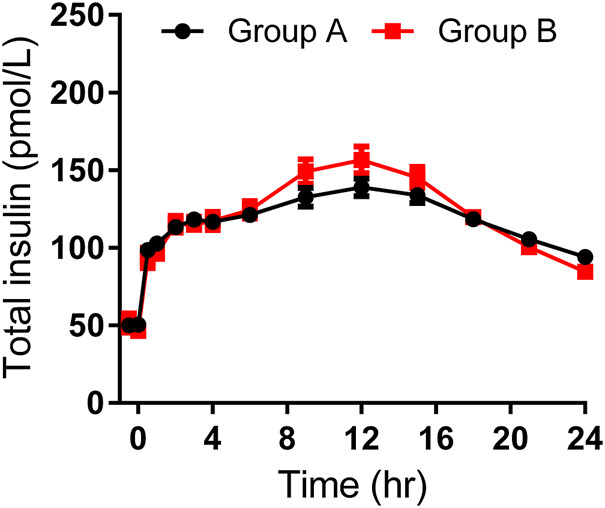
Time-profiles of observed total insulin throughout the euglycemic clamp in groups A and B, respectively (Mean ± SEM).

**FIGURE 3:**
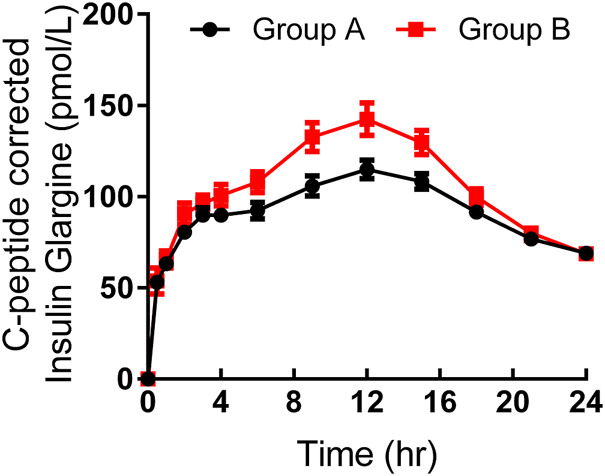
Time-profiles of insulin glargine derived from C-peptide correction throughout the euglycemic clamp in groups A and B, respectively (Mean ± SEM).

**FIGURE 4:**
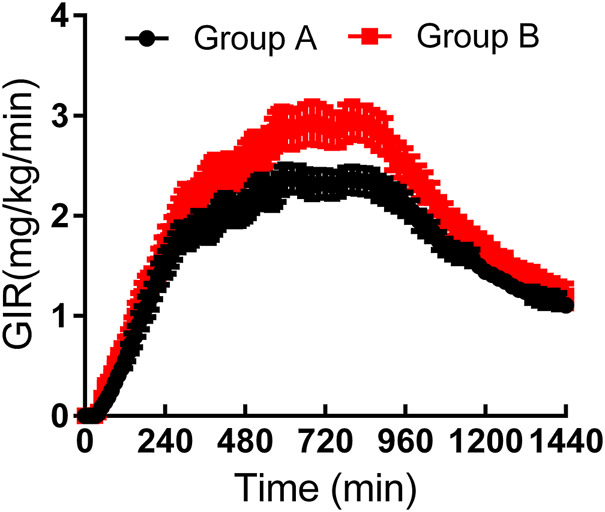
Time-profiles of glucose infusion rate throughout the euglycemic clamp in groups A and B, respectively (Mean ± SEM).

As shown in Table 3, the maximum observed total insulin and the AUC of total insulin from 0 to 12 or 24 h seemed to be slightly higher in group B than those in group A (Figure 2). The IGlar_max_, AUC_IGlar,0–12 h_, and AUC_IGlar,0–24 h_ were statistically higher in group B than those in group A (Figure 3). No difference was detected in tIGlar_max_. The observed GIR_max_ and AUC_GIR, 0–24 h_, were significantly higher in group A than those in group B, while tGIR_max_ was similar between the two groups (Figure 4). Since a significant difference existed in exogenous IGlar, which would contribute to the observed GIR, a correction for PD parameters using the corresponding exogenous IGlar parameter as a covariant was performed. After correction, the AUC_GIR, 0–24 h_ (2,697 vs*.* 2,513 mg/kg), GIR_max_ (3.09 vs*.* 2.96 mg/kg/min) and AUC_GIR, 0–24 h_ (1,256 vs*.* 1,213 mg/kg) were slightly higher in group B than those in group A.

**TABLE 3:** The pharmacokinetic and pharmacodynamic parameters in groups A and B.

	Group A (N = 52)	Group B (N = 26)	P
PD parameters			
AUC_GIR,0-12h_ (mg/kg) ^a^	989 (642)	1,320 (615)	0.015
AUC_GIR,0-24h_ (mg/kg) ^b^	2,415 ± 953	2,891 ± 908	0.038
GIR_max_ (mg/kg/min) ^b^	2.82 ± 1.06	3.36 ± 1.07	0.037
tGIR_max_ (min) ^b^	666 ± 202	678 ± 180	0.787
PK parameters			
AUC_IGlar,0-12h_ (pmol/L×h) ^b^	1,099 ± 342	1,295 ± 360	0.022
AUC_IGlar,0-24h_ (pmol/L×h) ^b^	2,194 ± 597	2,534 ± 597	0.020
IGlar_max_ (pmol/L) ^b^	122.7 ± 37.7	147.7 ± 42.5	0.010
tIGlar_max_ (h) ^b^	11.7 ± 3.97	11.6 ± 2.66	0.929
Other parameters			
AUC_Total insulin,0-12h_ (pmol/L) ^b^	1,444 ± 398	1,510 ± 368	0.482
AUC_Total insulin,0-24h_ (pmol/L) ^b^	2,853 ± 713	2,958 ± 626	0.524
Maximum total insulin (pmol/L) ^b^	151.8 ± 42.0	162.1 ± 41.9	0.310
Time of maximum total insulin (h) ^b^	10.9 ± 5.55	10.8 ± 3.77	0.937

GIR, glucose infusion rate; IGlar, insulin glargine; AUC, area under the curve.

a Median (interquartile range), the difference was detected by Mann Whitney test.

b Arithmetic mean ± standard deviation, the difference was detected by unpaired Student’s t-test.

### Odds Ratios of Factors for Sufficient Endogenous Insulin Suppression

The clamp quality, dose, and absorption of exogenous insulin were known factors affecting endogenous insulin inhibition. Throughout the clamp study, BG was closely maintained at the target level in both groups; therefore, CVBG was used to represent the quality of the clamp study. Binary logistic regression analysis was carried out with sufficient C-peptide reduction as a dependent variable, and age, basal BG, IGlar_max_, CVBG and AUC_IGlar,0–24 h_ as independent variables (Table 4). The *p*-value of the Hosmer and Lemeshow test was 0.584, indicating a successful establishment of the model. The results of the binary logistic regression analysis indicated that basal BG was an independent factor of sufficient endogenous insulin suppression (standardized odds ratio 0.010, *p* < 0.005).

**TABLE 4:** Odds ratios of factors for sufficient endogenous insulin suppression determined by logistic regression analysis.

	P	Standardized Odds Ratio	95%CI of Odds Ratio
**Age (year)**	0.151	0.788	0.569 to 1.091
**Basal BG (mmol/L)**	0.002	0.010	0.001 to 0.178
**CVBG (%)**	0.772	114,094	6.29 × 10^–30^ to 2.07×10^39^
**IGlar**_**max**_ **(pmol/L)**	0.386	1.020	0.975 to 1.068
**AUC**_**IGlar,0-24h**_ **(pmol/L×h)**	0.985	1.000	0.997 to 1.003

Confounding variables are listed in the panel. Variables were considered for the multivariable models when their univariable *p*-value was<0.10. Values are odds ratios with 95% confidence intervals (CI).

## Discussion

During the performance of a euglycemic clamp evaluating the PK/PD of a long-acting insulin analog, exogenous glucose infusion was frequently and properly adjusted according to the BG to ensure good quality (Heise et al., 2016; Kuhlenkotter et al., 2017; Benesch et al., 2022). Blood insulin concentration over time (PK) always predicts insulin action; in return, the GIR over time reflects the clinical glucose-lowering effect. However, the situation is ideally established based on the foundation that blood insulin concentration derives exclusively from absorption of the subcutaneously injected insulin and no endogenous insulin contributes to the observed GIR, e.g., a condition in patients with T1DM (Kerner et al., 1991; Porcellati et al., 2015). The effect of endogenous insulin on PK and/or PD may not be completely eliminated even with the use of a mathematical correcting method (Porcellati et al., 2015). Many clamp studies have been conducted in healthy people (Shiramoto et al., 2021; Linnebjerg et al., 2020b; de la et al., 2016) to meet regulatory requirements (i.e., to support the registration of a new drug by a regulatory body such as the EMA) and as a first indication of what to expect in diabetic patients (Food And Drug Administration, 2014). Therefore, sufficient endogenous insulin suppression of healthy volunteers is a key for a clamp with high quality (Kerner et al., 1991; Morris et al., 1997; Heise et al., 2016). C-peptide, which is secreted equimolecular to endogenous insulin by pancreas islets (Rubenstein et al., 1969), is usually regarded as an indicator of endogenous insulin. Many factors [e.g., individual metabolic differences, delayed blood monitoring (Bequette, 2009; Kuroda et al., 2017), investigator’s experience, and a several-minute lag between glucose reading and subsequent glucose infusion (Porcellati et al., 2011)] could affect the promptness and validity of GIR adjustment relating to the C-peptide suppression. In this article, we primarily aimed to explore how to achieve sufficient endogenous insulin suppression in euglycemic clamps evaluating the PK/PD of a long-acting insulin analog, and secondarily to assess the effect of the different endogenous insulin suppression on PK/PD assessment.

It is well accepted that a postdose C-peptide reduction of more than 50% from baseline indicates sufficient endogenous insulin suppression. The logistic regression analysis indicated low basal BG as an independent factor accounting for the success of sufficient endogenous insulin suppression. In addition, a much stronger C-peptide reduction was observed in clamp studies with a target BG set as 9 mg/dl below the baseline (Bhatia et al., 2018) compared to those whose target BG was set as 5 mg/dl below the baseline (Sorensen et al., 2010). The EMA (European Medicines Agency, 2015) suggests that stimulation of endogenous insulin secretion can be prevented by clamping at a sub-fasting glucose level, or by means of a continuous i. v. infusion of insulin throughout the clamp. No data suggest how to determine a sub-fasting glucose level, and different studies reported different selections [e.g., 5 mg/dl (Ponchner et al., 1984; Woodworth et al., 1994a; Howey et al., 1994; Arnolds et al., 2010; Sorensen et al., 2010; Leohr et al., 2020), 9–10 mg/dl (Thow et al., 1990; Woodworth et al., 1994b; Ter Braak et al., 1996; Hompesch et al., 2021) below the individual’s baseline]. It was observed that maintaining the BG at 5 mg/dl below the baseline was capable to achieve sufficient C-peptide suppression in group B where the mean basal BG was lower than 4.40 mmol/L, while a target BG set as 5 mg/dl below the baseline whose overall mean value was around 4.60 mmol/L was related to insufficient endogenous insulin suppression in group A. The increase in BG is known to motivate the entry of Ca^2+^ into the pancreatic β-cell to stimulate the release of insulin (Ozawa and Sand, 1986). Above-baseline of BG fluctuation was much less observed when BG was maintained around a lower sub-baseline level, therefore indicating a low possibility of stimulation of endogenous insulin (Liu et al., 2022). The phenomenon that insulin could inhibit its own secretion in normoglycemia was observed in many studies (Liljenquist et al., 1978; Service et al., 1978; Waldhaus et al., 1982; Bratusch-Marrain and Waldhausl, 1985). Additionally, suppression of C-peptide was observed in 80% of cases when BG was kept at a low-normal level (50–60 mg/dl) (Wasada et al., 1996). The inhibition of endogenous insulin relies on the glucose-mediated feedback, and this might be prior to an exogenous hyperinsulinemia-mediated inhibition (Wasada et al., 1996). Based on the findings of this study, we suggest that the target BG might be set at a lower level (e.g., 10% instead of 5% below the baseline) in a clamp with a higher basal BG (e.g., over 4.60 mmol/L) aiming to achieve sufficient endogenous insulin suppression.

After administration of an equal dosage of LY2963016 and Lantus, the C-peptide corrected IGlar showed a higher peak level and AUC_0–24 h_ of insulin concentration in group B. However, we think there existed a bias resulting in the invalidation of these results. Before C-peptide correction, the peak insulin and AUC of measured insulin from 0 to 24 h were slightly lower in group A, where endogenous insulin was not less sufficiently inhibited, which was not consistent with common sense. This might be due to a higher pharmacokinetic variety of IGlar and a limited sample size. A comparison between human insulin in parallel to the C-peptide measured by a specific assay and endogenous insulin calculated by C-peptide might elucidate the effect of different extents of C-peptide on PK estimates. The GIR_max_ and AUC_GIR,0–24h_ were still higher in group B before and after correction of exogenous IGlar. It was contradictory to what should be in theory. Although no significant difference was detected in HOMA-IR between the two groups, we still speculate a higher insulin sensitivity in group B because of a lower basal BG accompanied by a comparable basal insulin level. It remains uncertain whether a woman’s insulin sensitivity varies to such an extent that relevant changes in the experimental results may occur, depending on the point of time in her menstrual cycle (Toth et al., 1987; Diamond et al., 1989). A larger proportion of female participants in group A might be another reason for the confusing results.

This was a retrospective study including a total of 114 subjects. After the balance of Latus dosage, only a total of 26 subjects were allocated to group B. Limited sample size might affect the validation of the results. Further work with a larger sample size and a better design (i.e., only including males) will go straight to ascertain the way of setting a target BG and explore the effect of different C-peptide reductions on PK/PD assessment.

In conclusion, setting a lower sub-baseline target BG (e.g., 10% instead of 5% below the baseline) in a euglycemic clamp with a higher basal BG (e.g., higher than 4.60 mmol/L) might be an approach to help achieve sufficient endogenous insulin suppression.

## Data Availability

The original contributions presented in the study are included in the article/Supplementary Material, further inquiries can be directed to the corresponding author.
